# Supplementary material for: Potential roles of neuronal nitric oxide synthase and the PTEN-induced kinase 1 (PINK1)/Parkin pathway for mitochondrial protein degradation in disuse-induced soleus muscle atrophy in adult rats
Source: PLoS One. 2020 Dec 9;15(12):e0243660. doi: 10.1371/journal.pone.0243660 (PMC7725317; doi:10.1371/journal.pone.0243660)
Supplement: S1 Raw images — (PDF) [file pone.0243660.s001.pdf]

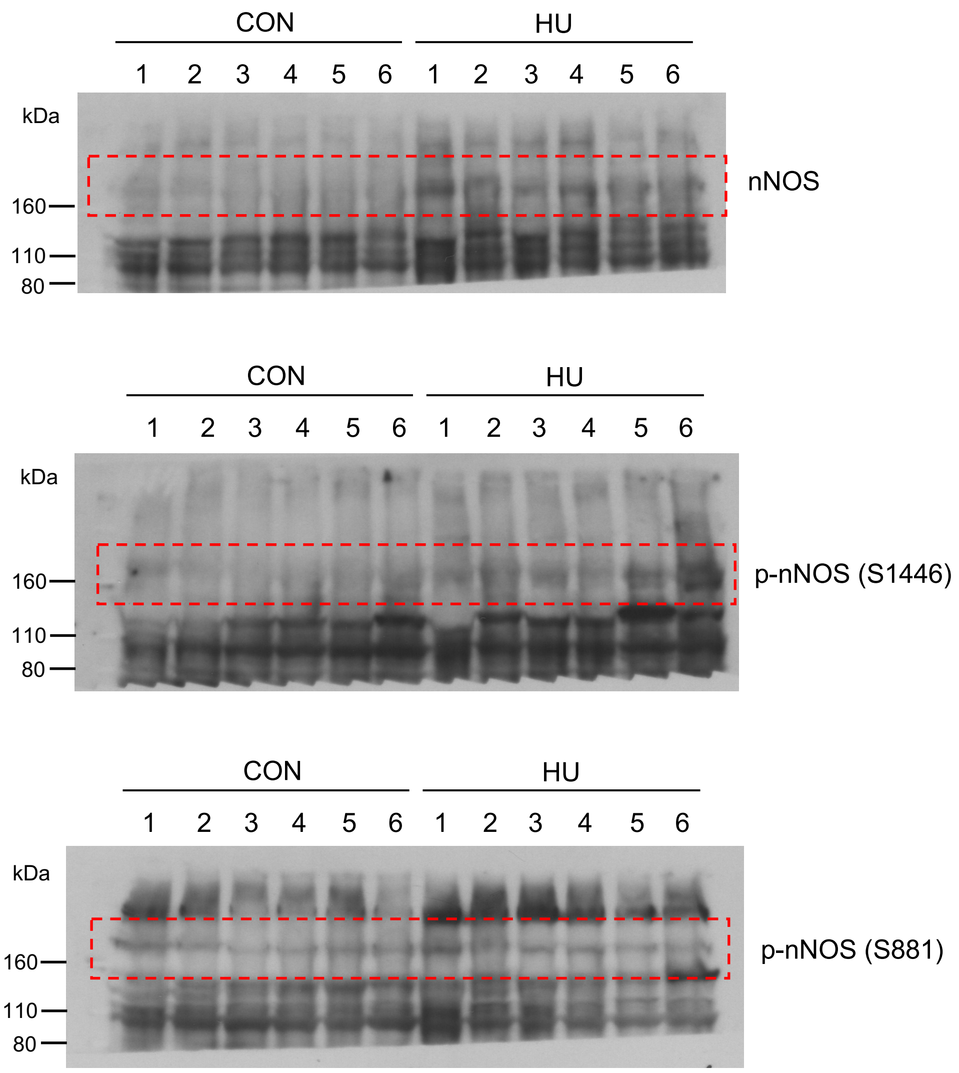

S1_raw_images. Original uncropped blots used in Fig. 2A.

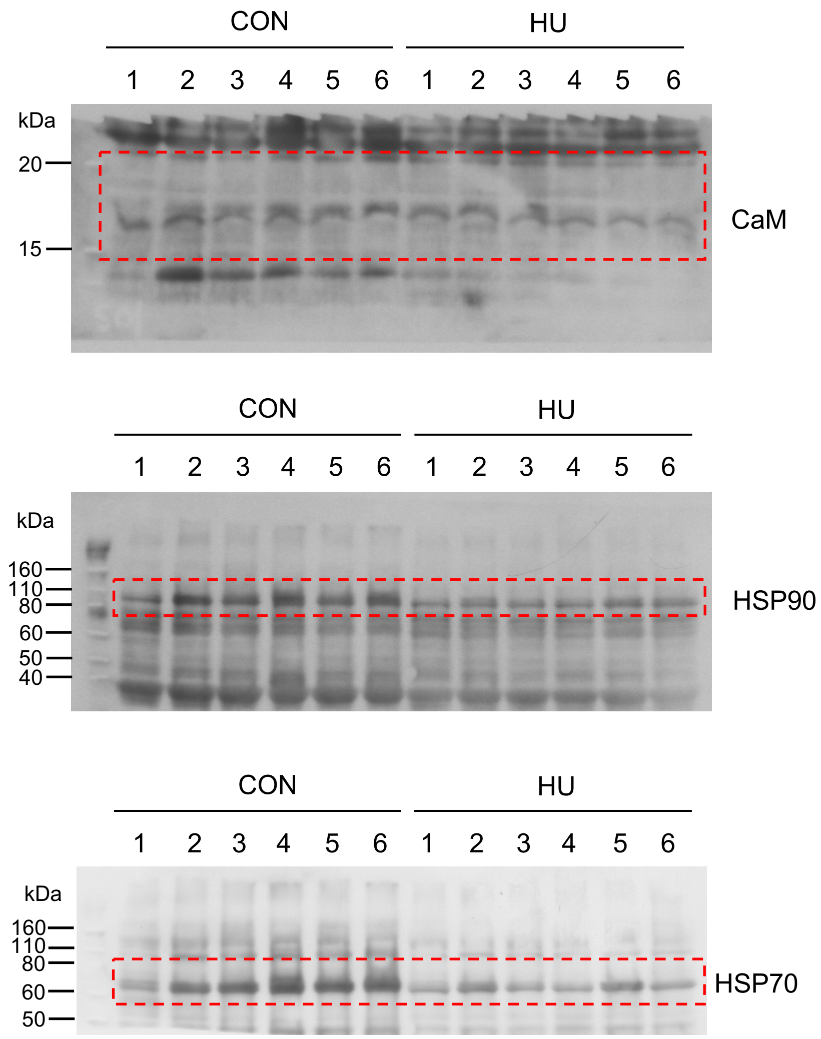

S1_raw_images. Original uncropped blots used in Fig. 3A.

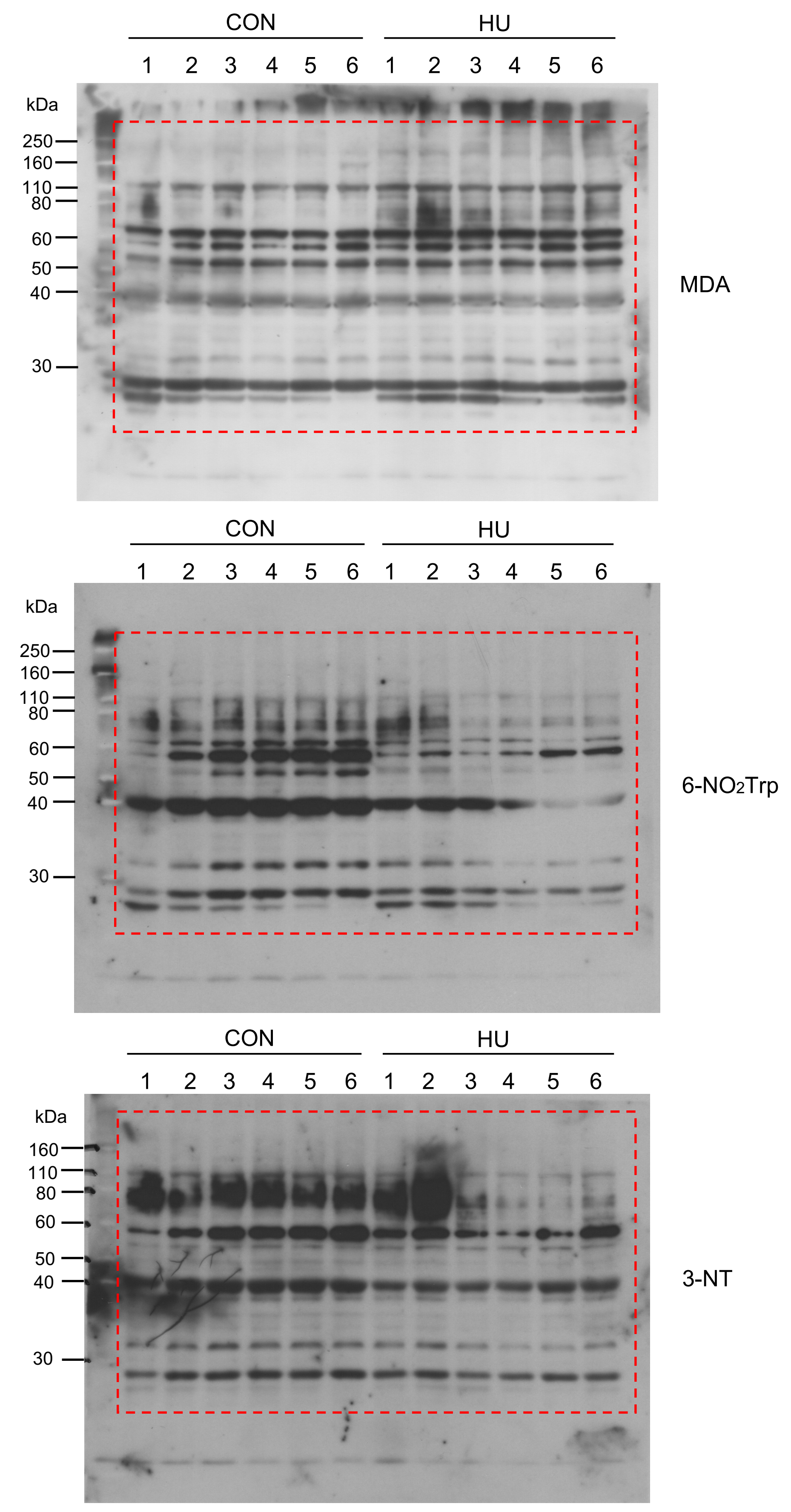

S1_raw_images. Original uncropped blots used in Fig. 4A-C.

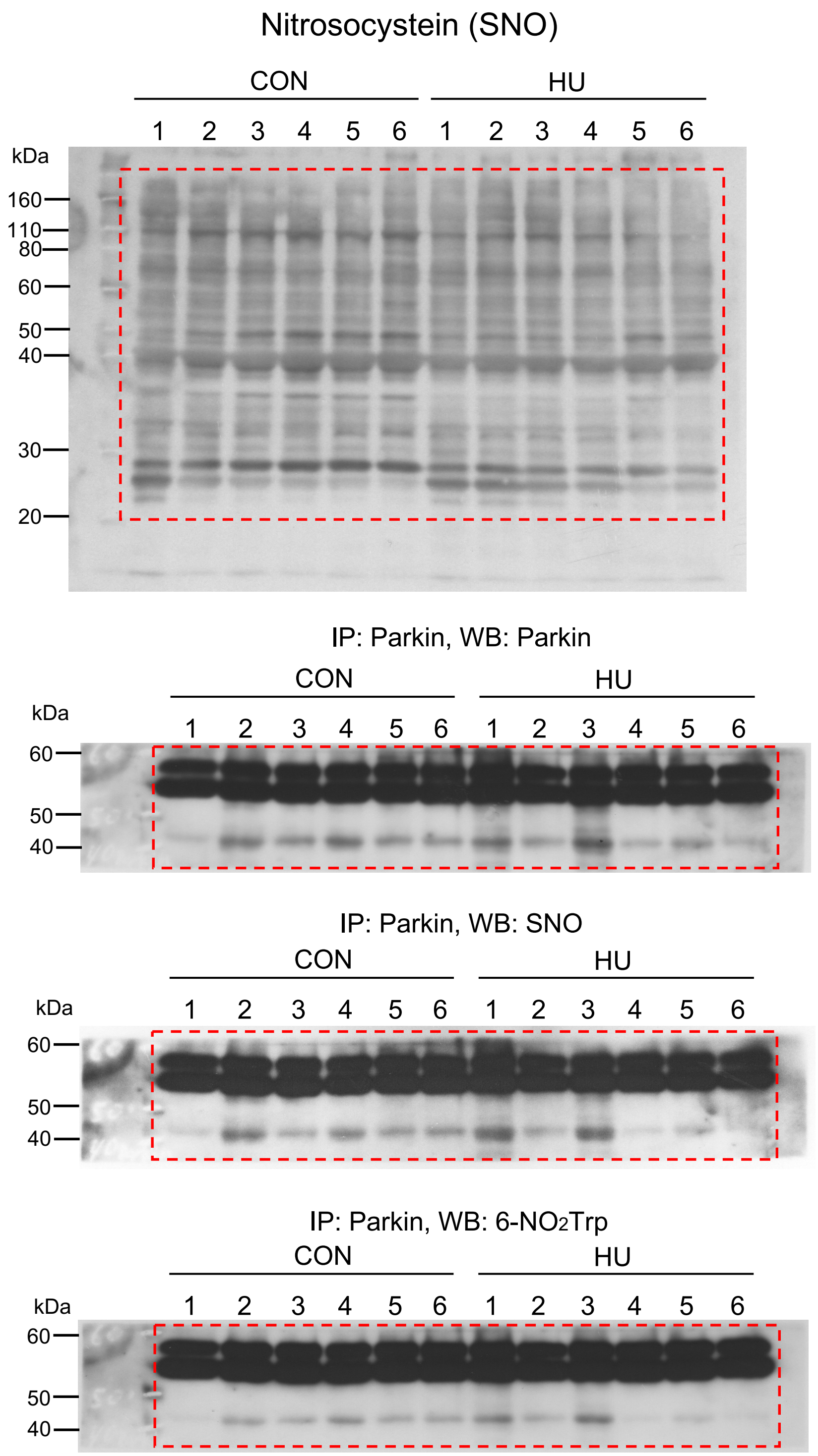

S1_raw_images. Original uncropped blots used in Fig. 5A and C.

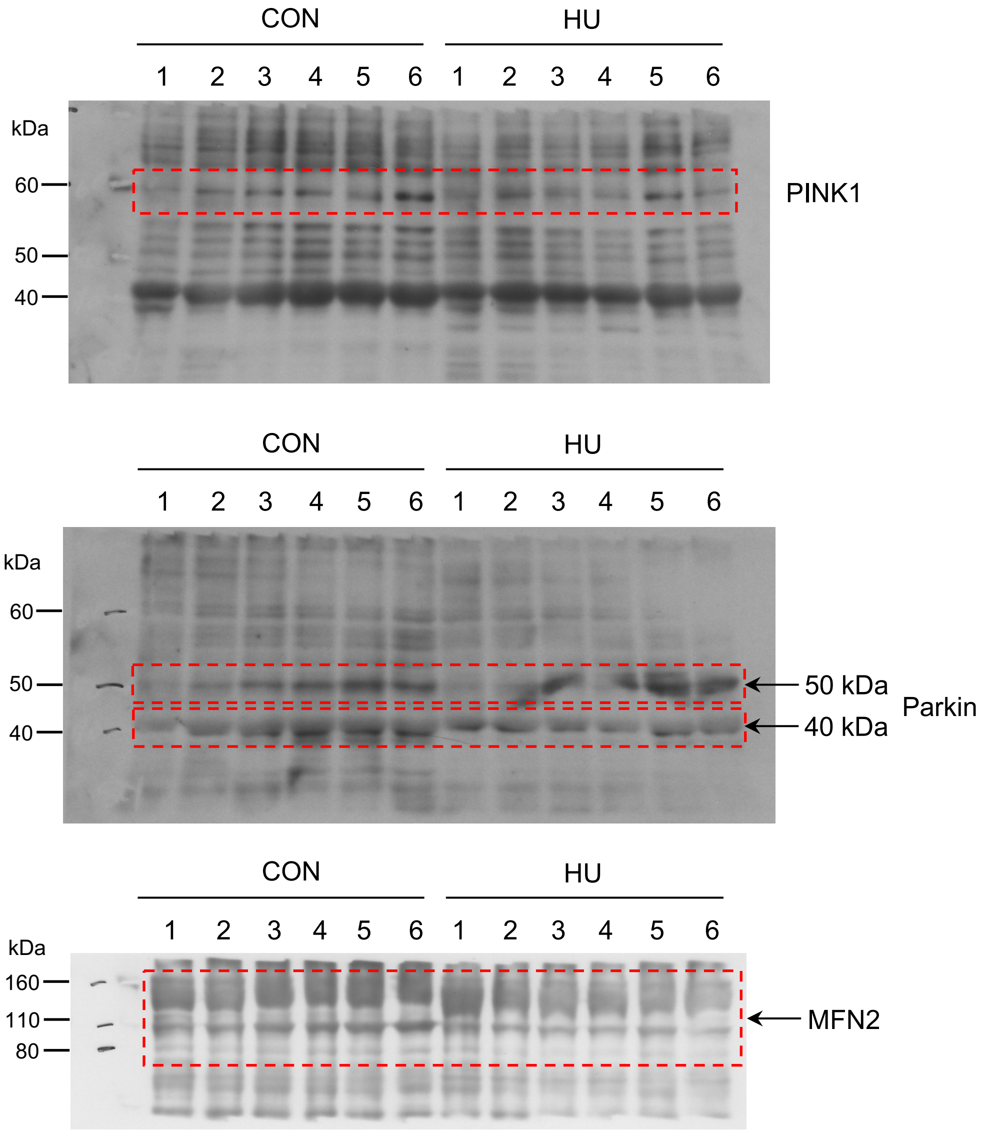

S1_raw_images. Original uncropped blots used in Fig. 6A.

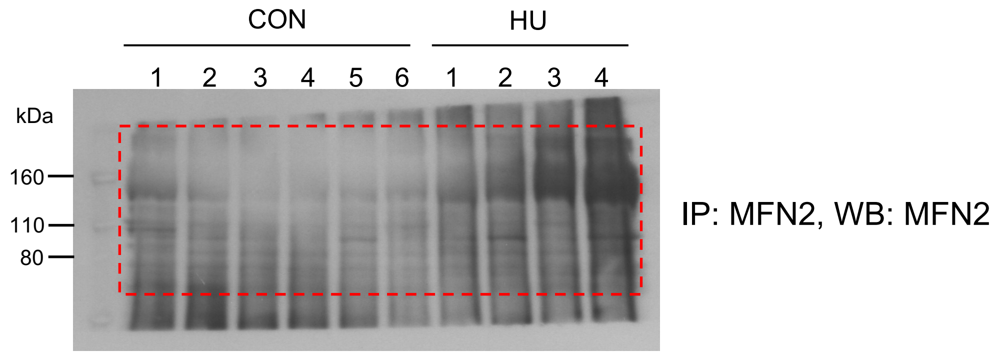

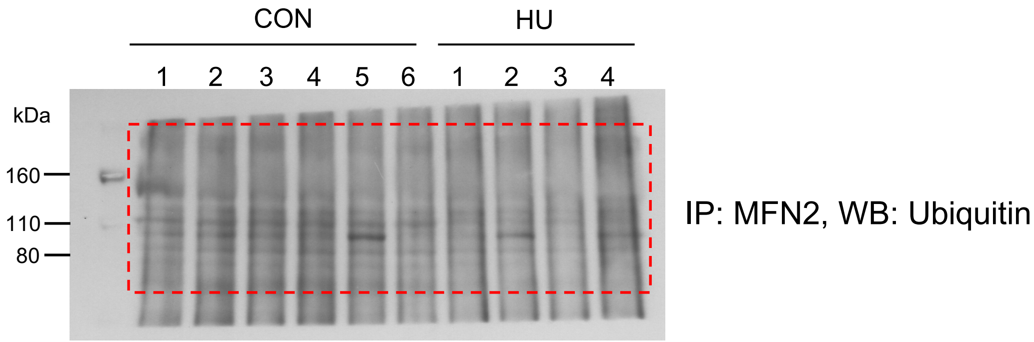

S1_raw_images. Original uncropped blots used in Fig. 7A.
